# Burden and Treatment Preference of Probable Neuropathic Pain in 13 592 people: A Cross‐Sectional and Discrete Choice Experiment in the General Population in Malaysia

**DOI:** 10.1111/jns.70154

**Published:** 2026-08-04

**Authors:** Wee‐Kiat Tan, Maznah Dahlui, Azhari Rosman, Hiang Leng Tan, Noraidah Guntalib, Siew Pheng Chan, Shalini Sree Dharan, Pei Chiek Teh, Daniel Yi Jie Wong, Kenny Tan, Solomon Tesfaye, Lee‐Ling Lim

**Affiliations:** ^1^ Department of Medicine Faculty of Medicine, Universiti Malaya Kuala Lumpur Malaysia; ^2^ Department of Social and Preventive Medicine Faculty of Medicine, Universiti Malaya Kuala Lumpur Malaysia; ^3^ National Heart Institute Kuala Lumpur Malaysia; ^4^ Lam Wah Ee Hospital Penang Malaysia; ^5^ Penang Adventist Hospital Penang Malaysia; ^6^ Subang Jaya Medical Centre Subang Jaya Selangor Malaysia; ^7^ Pantai Hospital Kuala Lumpur Kuala Lumpur Malaysia; ^8^ Damansara Specialist Hospital 2 Kuala Lumpur Malaysia; ^9^ Perak Community Specialist Hospital Ipoh Malaysia; ^10^ Loh Guan Lye Specialists Centre Penang Malaysia; ^11^ Division of Clinical Medicine University of Sheffield Sheffield UK; ^12^ Diabetes Research Unit Sheffield Teaching Hospitals NHS Foundation Trust Sheffield UK; ^13^ Department of Medicine and Therapeutics The Chinese University of Hong Kong Hong Kong SAR China; ^14^ Asia Diabetes Foundation Hong Kong SAR China; ^15^ Baker Heart and Diabetes Institute Melbourne Victoria Australia

**Keywords:** anti‐neuropathic agents, discrete choice experiment, neuropathic pain, peripheral neuropathy, treatment preference

## Abstract

**Background and Aim:**

Neuropathic pain (NeuP) is widely underdiagnosed, and treatment‐related adverse events often drive nonadherence. We investigated the probable NeuP prevalence, symptoms recognition, and treatment preferences among the general population in Malaysia.

**Methods:**

We utilized a digital ID‐Pain questionnaire across nine tertiary hospitals to screen for probable NeuP. We also designed a discrete choice experiment (DCE) to assess participants' ability to differentiate NeuP from nociceptive symptoms and to evaluate treatment preferences across five attributes (perceived pain reduction, time to effect, proven effectiveness, risk of common side effects, and cost) among the general population.

**Results:**

The screening cohort included 13 592 adults (17.1% with diabetes). The overall prevalence of screen‐positive for probable NeuP was 18.3% in the general population, increasing to 40.1% among people with diabetes. In the DCE cohort (*n* = 3999), 82.1% of participants demonstrated poor‐to‐moderate NeuP symptom recognition. Regarding treatment preferences, avoiding a high risk of common side effects was the primary driver of choice (coefficient −1.744, 95% CI −1.820, −1.669), followed by the desire for strong scientific evidence (coefficient 1.237, 95% CI 1.182, 1.291). Apart from clinical attributes, participants showed significant cost sensitivity (coefficient −5.565, 95% CI −6.909, −4.221).

**Interpretation:**

Despite a high prevalence of probable NeuP, poor symptom recognition creates a significant diagnostic challenge. Given that aversion to side effects is prioritized over other attributes, clinicians need to focus on tolerable anti‐neuropathic agents and employ proactive side‐effect management to optimize NeuP treatment adherence.

## 1 Introduction

The global disease burden of polyneuropathy among the adult population is estimated at 7%–10% [1, 2]. As polyneuropathy progresses, some people may develop painful sensation [3]. Neuropathic pain (NeuP) is defined as pain sensation that arises from a lesion in the somatosensory system [4]. NeuP is broadly classified into acute (≤ 3 months) and chronic pain (> 3 months) [5]. The leading causes of NeuP worldwide are painful diabetic polyneuropathy and postherpetic neuralgia [6]. Around half of the people with diabetes [7] and 20% of herpes zoster infections lead to NeuP [8].

There are gaps in NeuP care; up to 50% of patients with NeuP were undiagnosed [9]. Delayed diagnosis and management of acute NeuP lead to chronic NeuP, which is associated with increased risks of anxiety and depression by 60% [10], and coronary artery disease, with an odds ratio of 2.27 (95% CI 1.21, 4.60) [11]. Unfortunately, up to 34% of patients are nonadherent to pain medications [12], in which their perspectives are not fully understood.

Understanding the perspectives and preferences of the community is essential in designing optimal care delivery for chronic conditions, including NeuP [13]. Discrete choice experiment (DCE) is an emerging methodology to elicit the perspectives and preferences of stakeholders in health research [14]. We can utilize DCE to investigate stakeholders' preferences for various attributes and measure the trade‐offs between them [14]. A DCE identifies the preferred attributes from the care receivers' perspectives, ensuring that the care design reflects the needs and priorities of the people we serve. In the present study, we assessed the burden of probable NeuP using a community survey while evaluating treatment preferences using the novel DCE approach among the general population in Malaysia.

## 2 Materials and Methods

### 2.1 Study Design and Participants

This study utilized a cross‐sectional design incorporating two complementary data collection approaches to assess both the burden of probable NeuP and treatment preferences in the general population. First, to quantify the burden of probable NeuP among people aged 18–90 years (inclusive), we conducted a community‐based digital survey using self‐administered electronic data capture. We installed 10 light‐emitting diode (LED) touchscreen kiosks (61.7 cm × 178 cm) with internet connectivity at nine tertiary hospitals nationwide to facilitate large‐scale recruitment (Figure S1). Each kiosk hosted a progressive web application containing the six‐item ID‐Pain questionnaire (Figure S2). The ID‐Pain questionnaire, which has previously been validated [15, 16], is easy to understand and recommended as a screening tool by the International Association for the Study of Pain [17]. To optimize feasibility and maximize recruitment, the screening instrument was streamlined to have three additional demographic questions, namely age group (18–30, 31–40, 41–50, 51–60, and > 60 years), gender (men and women), and the status of self‐reported diabetes (yes, no, and unknown). No personally identifiable information was collected. Responses were automatically scored, generating values ranging from −1 to 5. An ID‐Pain score ≥ 3 was classified as screen‐positive for probable NeuP [18]. To evaluate the representativeness of the community‐screening cohort relative to the general population in Malaysia, the demographic distribution of participants was cross‐referenced against national demographic indicators and diabetes burden (Table S1) [19, 20]. A cutoff of 10% deviation was set for appropriate representation [21].

Second, we designed a DCE to evaluate the ability to recognize the symptoms of NeuP and treatment preferences among the general population. We designed a structured set of test questions comprising 13 pain descriptors, including nine neuropathic descriptors (pins and needles, hot or burning, numbness, electric‐shock, pain worsened by light touch, for example, clothes or bed sheets, painful cold, itching, tingling, and insensitivity to touch) and five common nociceptive descriptors (dull pain, sharp pain, aching, throbbing, and tenderness), with additional response options of “all of the above” or “none of the above.”

We derived the neuropathic descriptors from established NeuP screening instruments, namely ID‐Pain [18], PainDETECT [22], and Douleur Neuropathique‐4 (DN4) [23]. We set a predefined algorithm for the scoring to reflect both identification and discrimination ability for neuropathic and nociceptive symptoms [24]. For neuropathic descriptors, we set the scores of 1, 2, and 3 for correctly recognizing 1–3, 4–6, and 7–9 symptoms, respectively. For nociceptive descriptors, reverse scoring was used, with scores of 3, 2, and 1 assigned for correctly recognizing 1–2, 3, and 4–5 symptoms, respectively. Following that, we calculated a composite recognition metric [25] by subtracting the nociceptive score from the neuropathic score, with higher values indicating greater ability to correctly recognize and differentiate the symptoms of NeuP. Three levels of ability to recognize NeuP symptoms were defined, namely poor, moderate, and good.

For preference elicitation, we identified the attributes of NeuP treatment through a systematic review of preference studies on pain and refined them through consultation with the relevant experts to ensure clinical relevance and cultural appropriateness within the Malaysian context [26]. We finalized five attributes: (1) perceived pain improvement (percentage reduction in pain), (2) time to effect (weeks to meaningful relief), (3) proven effectiveness (strength of supporting scientific evidence), (4) risk of common side effects (e.g., dizziness and somnolence), and (5) cost per tablet (price in Malaysian Ringgit [MYR]) (Table S2). To facilitate questionnaire completion, we provided the standardized definitions of all attributes to the study participants. The example of a choice task is shown in Table S3.

Using Ngene software, we generated a pilot DCE questionnaire with a Bayesian efficient design with standardized priors [27] with balanced alternatives (no dominance in option). We tested the pilot instrument among 45 participants without a healthcare background to evaluate comprehension and derive preliminary coefficients to inform the experimental design of the questionnaire. The clarity of the questionnaire (namely the sentence structure and choice of words) was also improved with participants' feedback from the pilot phase. The final DCE questionnaire comprised 12 choice tasks per participant, requiring participants to make trade‐offs between hypothetical treatment options. We used these data to quantify the relative utility of treatment attributes and estimate stated treatment preferences. To ensure data quality, we incorporated a dominance test and masked it from the questionnaire administrator. We also excluded data from participants who completed the questionnaires in less than 30 s, which was the cutoff established based on the minimum completion duration observed in the pilot instrument.

### 2.2 Sample Size Calculation

For the community‐based screening component, large‐scale recruitment was facilitated through digital interfaces deployed at eight tertiary hospitals over 24 months (December 2023 to December 2025). Based on the expected duration of deployment and site throughput, we targeted a minimum of 7000 completed responses to provide reliable estimates of the prevalence and pre‐specified subgroup analysis by age group, gender, and the status of self‐reported diabetes.

For the DCE component, the reported prevalence of NeuP in the general population is estimated to be around 10% [2]. Using a type 1 error probability of 5% and at a 95% confidence level (CI), the minimum sample size was 390 people with probable NeuP in this cross‐sectional DCE [28]. Taking 10% as the prevalence of NeuP among the general population, the minimum target recruitment was 3900 participants. This ensured an adequate number of affected participants for symptom recognition analysis and preference modeling in the DCE.

### 2.3 Ethics Statement

This study was approved by the Medical Research Ethics Committee (MREC), Universiti Malaya Medical Centre, Kuala Lumpur, Malaysia (ID: 2025530‐15131).

### 2.4 Statistical Analysis

We conducted all analysis using de‐identified data obtained from the automated community‐based screening platform and DCE questionnaire. For the community‐screening component, we presented the frequency of the symptoms of NeuP as count (N) and percentage (%). We determined the likelihood of NeuP using the ID‐Pain scoring system [18], where the presence of each positive symptom contributed one (1) score and minus one (1) score if the pain was limited to the joint only. The likelihood of NeuP increases with a higher total score. Probable NeuP was considered if the total score was ≥ 3. We calculated the prevalence of screen‐positive for probable NeuP in the general population by dividing the number of participants who scored ≥ 3 by the total number of completed responses and presented as a percentage.

For the DCE component, we analyzed the findings with a multinomial model (MNL) and a mixed logit model (MXL) for main effects and the interactions. To compare the relative utility ratio between attributes and their levels, we set the high risk of side effects (10%) as the reference. We also calculated the marginal substitution rate of respective attributes against the coefficient of high risk of side effects to measure tradeoffs between attributes. We selected the best model based on the goodness‐of‐fit criteria, including the likelihood ratio (LR) test, Akaike Information Criterion (AIC), and Bayesian Information Criterion (BIC) [29]. Lower AIC and BIC and significant LR improvements indicate a superior fit. All analyses were conducted using the Apollo package in R software (Supporting Information S1) [30].

## 3 Results

### 3.1 Participant Characteristics

A total of 13 592 participants completed the community‐based digital screening. Another 4153 participants completed the DCE, excluding the data from participants who failed the dominance test and demonstrated irrational behavior; 3999 participants were included in the final analysis. Table 1 summarizes the participant characteristics. In the community‐screening cohort, 23.1% of participants aged 18–30 years, 47.8% were men, and 17.1% had self‐reported diabetes status. In the DCE cohort, 27.1% of the participants aged 31–40 years, 48% were men, 59.1% received tertiary education, and 22.5% reported a monthly household income of MYR 5000–8000. One fifth of participants had prior history of either hypertension or hypercholesterolemia, and 9.7% had self‐reported diabetes status.

**TABLE 1:** Participants' characteristics of the community screening and DCE cohorts.

Characteristics		Community‐screening cohort (*N* = 13 592)		DCE cohort (*N* = 3999)	
		*n*	%	*n*	%
Age group	18–30	3135	23.1	1034	25.6
	31–40	2785	20.6	1084	27.1
	41–50	2746	20.2	1005	25.1
	51–60	2243	16.5	503	12.6
	61–70	2673	19.7	309	7.7
	71–80			61	1.5
	> 80			3	0.1
Gender	Men	6493	47.8	1919	48.0
	Women	7099	52.2	2080	52.0
Education level	Postgraduate	NA	NA	330	8.3
	Degree	NA	NA	2008	50.2
	Diploma	NA	NA	914	22.9
	Secondary education	NA	NA	708	17.7
	Primary education	NA	NA	25	0.6
	No formal education	NA	NA	14	0.4
Household income per month	Less than MYR 3000	NA	NA	594	14.9
	MYR 3000–5000	NA	NA	848	21.2
	MYR 5000–8000	NA	NA	900	22.5
	MYR 8000–10 000	NA	NA	571	14.3
	MYR 10000–15 000	NA	NA	586	14.7
	More than RM 15 000	NA	NA	386	9.7
Comorbidities	Hypertension	NA	NA	841	21.0
	Hypercholesterolemia	NA	NA	869	21.7
	Diabetes mellitus	2331	17.1	390	9.7
	Heart disease	NA	NA	62	1.6
	Eye disease	Yes	NA	NA	114
	Stroke	NA	NA	24	0.6

*Note:* Heart disease was defined by the presence of any coronary heart disease(s). Eye disease was defined by the presence of any condition(s) that affect the vision or the structure of the eye. The comorbidities were self‐reported. Household income per month classifications were defined according to the income distribution in the Household Income Survey Report 2024 by the Department of Statistics, Malaysia.

Abbreviations: *N*, number of participants; NA, not available.

### 3.2 Community‐Screening Cohort

#### 3.2.1 Prevalence of Screen‐Positive for Probable NeuP

Among 13 592 participants, 2493 (18.34%) reported having probable NeuP. Within 2331 participants with diabetes, 935 (40.11%) of them had probable NeuP, compared with 1558 (13.84%) among those without diabetes (Table 1). The three most frequently reported symptoms were identical for participants across subgroups with probable NeuP: pins and needles, numbness, and electric‐shock sensations. Among participants with probable NeuP, pins and needles sensation was the most prevalent (91.21%–96.04%), followed by numbness (89.20%–93.90%) and electric‐shock sensations (80.74%–87.21%). By contrast, for participants with unlikely NeuP, numbness predominated (26.05%–44.27%), followed by pins and needles (19.12%–35.74%) and electric‐shock sensations (16.73%–18.94%). These were consistent across the subgroups by age group, gender, diabetes status, and geographical regions in Malaysia (Table S4).

### 3.3 DCE Cohort in the General Population

#### 3.3.1 Recognition of Probable NeuP Symptoms

In the DCE cohort, 2201 (55%) showed moderate ability to recognize probable NeuP symptoms, while 715 (17.8%) showed good ability. A total of 1083 (27.2%) of them demonstrated poor ability (Table S5).

#### 3.3.2 Treatment Preferences of NeuP

**FIGURE 1:**
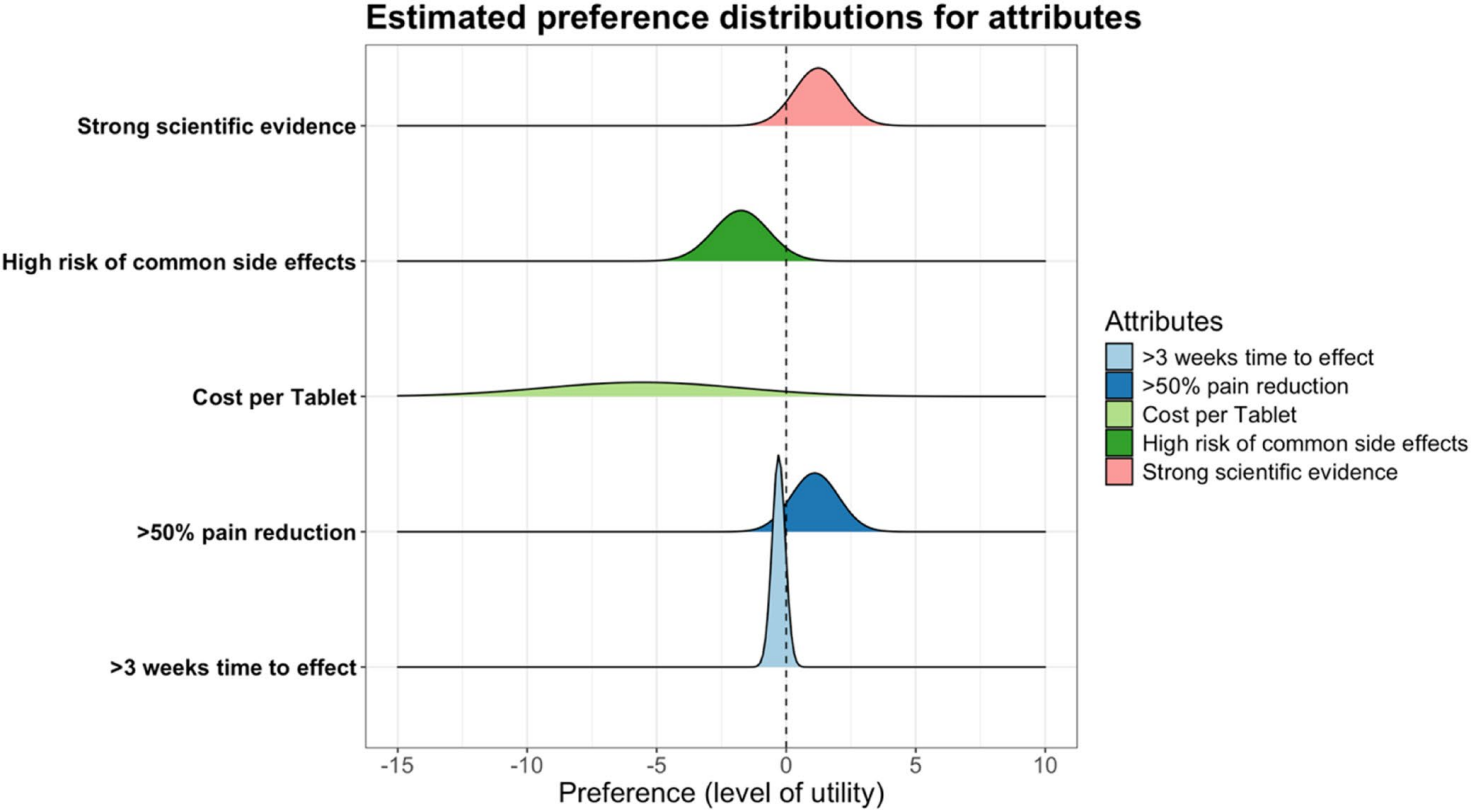
The density plot of NeuP treatment preference distribution at the highest level of attributes in DCE. Distribution to the right shows a positive utility (preferred), whilst distribution to the left represents disutility (not preferred) of NeuP treatment preference. The height and width of the plot represent the distribution of treatment preference, that is, a narrow and tall plot shows a concentrated distribution (low heterogeneity), whilst a wide and short plot shows a diverse distribution (high heterogeneity). DCE, discrete choice experiment.

Table 2 shows the main effects from the mixed logit model. The risk of common side effects, time to effect, and cost impacted the utility negatively, while the level of scientific evidence and perceived pain reduction had a positive influence on the utility. Of all attributes, cost had the highest negative utility (coefficient −5.565, 95% CI −6.909, −4.221), followed by the high risk of common side effects (coefficient −1.744, 95% CI −1.820, −1.669). The > 3 weeks' time to effect attribute (coefficient −0.290, 95% CI −0.332, −0.249) had the lowest magnitude among attributes with negative utility. Strong scientific evidence had the highest positive utility (coefficient 1.237, 95% CI 1.182, 1.291), followed by > 50% perceived pain improvement (coefficient 1.099, 95% CI 1.041, 1.157). Apart from the cohort average as shown in the main effects model, we also explored heterogeneity of preference. Preference heterogeneity was identified only at the highest level of all attributes. Cost showed substantial heterogeneity, while time to effect had the lowest heterogeneity, as shown in Figure 1.

**TABLE 2:** Main effects of attributes from the mixed logit model in the DCE model.

Attributes	Levels	Coefficient	95% CI
Cost	Cost (continuous)	−5.565	−6.909, −4.221
Perceived pain reduction	< 20%	Reference	—
	20%–30%	−0.038	−0.086, 0.010
	30%–50%	0.328	0.280, 0.376
	> 50%	1.099	1.041, 1.157
Time to effect	1–2 weeks	Reference	—
	2–3 weeks	−0.200	−0.246, −0.153
	> 3 weeks	−0.290	−0.332, −0.249
Level of scientific evidence	Limited scientific evidence	Reference	—
	Some scientific evidence	0.437	0.384, 0.490
	Strong scientific evidence	1.237	1.182, 1.291
Risk of common side effects	Low (0.001%)	Reference	—
	Moderate (0.01%)	−0.537	−0.583, −0.492
	High (10%)	−1.744	−1.820, −1.669

*Note:* A positive value represents a preferred attribute, while a negative value represents an unpreferred attribute. CI, confidence interval; DCE, discrete choice experiment.

**FIGURE 2:**
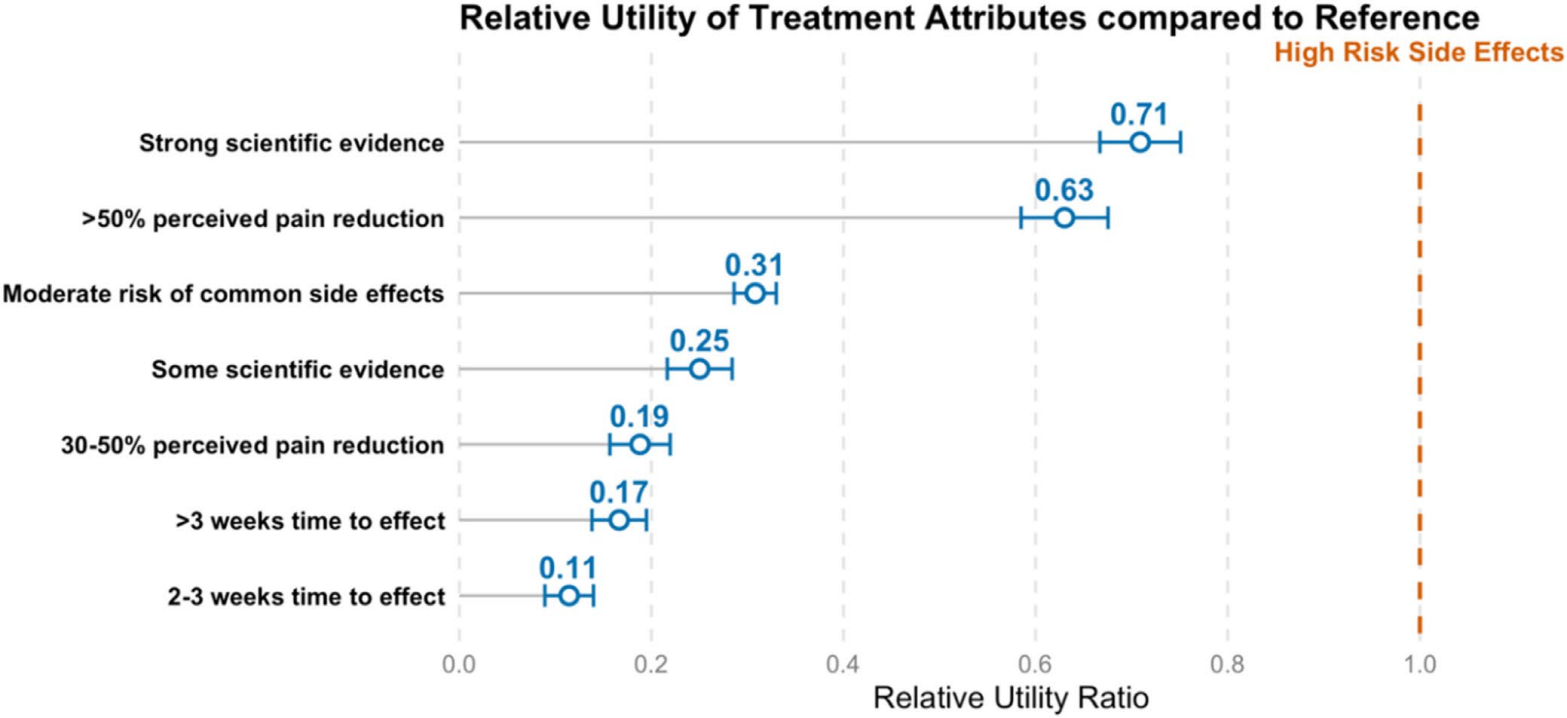
The relative utility of all levels of attributes, using high risk of common side effects as the reference attribute in DCE. The coefficient of high risk of common side effects (10%) was −1.744 (95% CI −1.820, −1.669), which was the largest magnitude of utility among the clinical attributes. Thus, it was set as a reference to compare the relative utility against other clinical attributes. Strong scientific evidence had the highest relative utility. DCE, discrete choice experiment.

Since the high risk of common side effects attribute had the highest magnitude of coefficient, it was set as the reference to measure the relative utility of other attributes. Figure 2 shows the relative utility of other attributes compared with the high risk of common side effects attribute. Strong scientific evidence and > 50% perceived pain reduction had the highest relative utility, whilst the time‐to‐effect attribute had the lowest relative utility. In terms of tradeoffs between attributes, participants were willing to accept the high risk of common side effects with a 1.58‐unit increase from > 50% perceived pain reduction (Table S6). Apart from the attributes' tradeoffs, willingness‐to‐pay was calculated. Compared with the respective reference level of the attributes, participants were willing to pay MYR 9.350 per tablet extra for achieving > 50% perceived pain reduction, MYR 11.630 for receiving treatment with strong scientific evidence, and MYR 18.662 for avoiding common side effects (Table S7).

#### 3.3.3 Heterogeneity in Preference

In the presence of heterogeneity, we quantified its magnitude through the interaction terms between attributes and participants' characteristics (Table S8). Interactions were observed for the education level, status of pain, age group, and monthly household income. Compared with those with a lower education level, participants with a higher education level had a higher preference for > 50% perceived pain reduction (coefficient 1.120, 95% CI 1.054, 1.187 versus coefficient 0.997, 95% CI 0.887, 1.106) and strong scientific evidence (coefficient 1.289, 95% CI 1.223, 1.354 versus coefficient 1.000, 95% CI 0.895, 1.106). However, participants with a higher education level showed a stronger aversion to a high risk of common side effects compared with the lower educated group (coefficient −1.799, 95% CI −1.890, −1.708 versus coefficient −1.586, 95% CI −1.743, −1.429). Besides, participants with a higher education level demonstrated a lower concern about cost than their lower educated counterparts (coefficient −5.040, 95% CI −6.219, −3.861 versus coefficient −5.980, 95% CI −7.273, −4.686). Compared with those without probable NeuP, participants with probable NeuP were less willing to tolerate a high risk of common side effects (coefficient: −1.811, 95% CI: −1.908, −1.714 versus coefficient: −1.631, 95% CI: −1.759, −1.503).

Interaction by age revealed that older participants placed a higher value on achieving > 50% perceived pain reduction compared to younger cohorts (age 51–70 years: coefficient 1.265, 95% CI 1.149, 1.380; age > 70 years: coefficient 1.549, 95% CI 1.153, 1.946) (Table S8). However, these older groups also showed a stronger aversion to a high risk of common side effects (age 51–70 years: coefficient −1.896, 95% CI −2.051, −1.741; age > 70 years: coefficient −2.511, 95% CI −3.065, −1.957). When analyzed by monthly household income, cost concern and preference for scientific evidence remained similar across all characteristics. In contrast, the disutility associated with a high risk of common side effects increased with household income level; the high‐income group exhibited the strongest aversion (coefficient −1.958, 95% CI −2.100, −1.816), followed by the middle‐income (coefficient −1.821, 95% CI −1.941, −1.701), and low‐income groups (coefficient −1.549, 95% CI −1.668, −1.430).

## 4 Discussion

People with NeuP are often underdiagnosed and undertreated [31], which may lead to devastating implications psychologically and physically. Given the dire situation, we sought to understand the burden of probable NeuP, symptom recognition ability, and treatment preference. Our findings showed an 18.3% prevalence of probable NeuP in the general population, with a higher rate of 40.1% among those with self‐reported diabetes. We also identified a concerning knowledge gap, whereby most participants demonstrated a poor to moderate ability to recognize NeuP‐specific symptoms (pins and needles, numbness, and electric‐shock sensations). In evaluating NeuP treatment preferences among the public, the risk of common side effects was identified as the primary driver of choice, followed by treatment effectiveness. Further analysis revealed that this aversion to side effects was significantly stronger among older participants, as well as those with higher education and household income levels. Notably, participants demonstrated a clear willingness to incur higher out‐of‐pocket costs to avoid treatment‐related side effects.

The prevalence of all‐cause probable NeuP observed in the present study was higher than that reported in other key community‐based studies. For instance, prevalence rates were 9.2% in the UK Biobank cohort [32], 9.8% in the United States [33], and only 3.2% in Japan [34]. These disparities may be due to differences in the demographics of study populations, assessment tools utilized (e.g., ID‐Pain, painDETECT, and DN‐4), and classifications of NeuP. However, among participants with self‐reported diabetes, our findings align with global estimates. The pooled global prevalence of NeuP in people with diabetes was 46.7% [7], with regional studies reporting rates of 57.2% in China [35], 43.3% in the Arabian Gulf region [36], and 34.0% in the United Kingdom [37]. Our findings are also consistent with a previous study in Malaysia, which reported painful diabetic peripheral neuropathy (DPN) symptoms in 41.3% of people [38]. Despite this high prevalence, painful DPN remains largely underdiagnosed at 60%–80% [39, 40], which is disproportionately higher than other noncommunicable diseases such as hypertension (28.6%) [41]. Alarmingly, untreated NeuP is strongly associated with pain chronification [42], which can lead to a deteriorating cycle of poor sleep and adverse mental health outcomes.

The underdiagnosis of NeuP is a multifaceted issue driven by both clinician‐ and patient‐related factors. The ability to recognize symptoms is a key determinant of treatment‐seeking behavior [24]. In the present study, we found that 82.1% of participants demonstrated poor to moderate ability to recognize NeuP‐specific symptoms. Our findings mirror the Malaysian national health survey, indicating that 75.7% of Malaysians have a poor to moderate level of general health literacy [43]. This lack of symptom recognition provides a plausible explanation for the underdiagnosis of NeuP. In real‐world practice, even competent clinicians face significant diagnostic challenges when patients are unable to accurately recognize and articulate their symptoms.

The clinical use of anti‐neuropathic agents is often hindered by resistance to initiating treatment, suboptimal adherence, and high discontinuation rates [44]. The present DCE evaluation sheds light on these behaviors by evaluating the treatment preference among the general population. Consistent with previous pain treatment preference studies, pain reduction effectiveness, the risk of side effects, and cost were the prioritized attributes. Our findings mirror a US‐based study which valued a low risk of side effects the most, followed by treatment effectiveness and cost [45]. In contrast, a United Kingdom cohort was primarily sensitive to cost, followed by effectiveness and side effects risks [46]. While the core priorities remain similar across studies, these sequential differences likely stem from divergent economic structures, sociocultural backgrounds, and the specific type of pain experienced. Notably, we reported that the strong aversion to common side effects was particularly pronounced among people who already experience NeuP, as well as those with higher education and higher income levels.

The struggle to balance pain reduction and adverse reactions is a common clinical dilemma in managing NeuP. In our tradeoff analysis, participants required pain reduction of 80% in exchange for a 10% risk of common side effects. However, standard anti‐neuropathic agents usually produce < 50% pain reduction [47]. This stark disconnection between participants' expectations and clinical reality provides a compelling explanation for the suboptimal adherence and high discontinuation rate of anti‐neuropathic agents.

To address the challenges, we propose three targeted strategies. First, healthcare facilities should establish dedicated NeuP screening touchpoints. Our experience suggests that automated self‐assessment interfaces could potentially encourage voluntary screening; the minimal resource requirements make them highly sustainable for large‐scale use. Second, education regarding NeuP symptoms must be enhanced among people with diabetes, as DPN is one of the most common causes of chronic NeuP. Empowering this high‐risk population to recognize early NeuP symptoms enables early interventions and prevents pain chronification [42]. Finally, clinicians must improve communication regarding side effects management when initiating anti‐neuropathic agents. Given that the present DCE shows that participants are highly reluctant to trade the risk of side effects for treatment effectiveness, clinicians must set proper expectations, emphasize the temporary nature of most side effects, the need for individualized titration schedule, and the employment of mitigation strategies, including asymmetrical dosing or bedtime administration, whenever appropriate.

To our knowledge, this is the first and largest study to investigate community preferences regarding anti‐neuropathic treatment attributes. By utilizing DCE approach, we successfully elucidated the participant‐level factors that likely contribute to suboptimal treatment outcomes of NeuP. Furthermore, the present study provides empirical demonstration for an innovative, automated self‐assessment method for probable NeuP screening, scoring its potential for large‐scale and sustainable community use.

However, the present study has several limitations. Firstly, although the community‐screening cohort largely resembled the national census data, participants of age group 31–40 years were under‐represented, which warrants cautious interpretation of disease burden within this specific age group. Second, the self‐reported assessments were susceptible to strategic responding, where participants answered with specific intentions that could skew the data. We mitigated this risk by having a large sample size in the community screening and DCE cohorts, as well as utilizing dominance test to confirm rational choice behavior in the DCE cohort. Third, the online administration of the DCE survey may have introduced selection bias, potentially excluding populations with limited digital literacy. As our study participants were the general population with varying burden of probable NeuP, the hypothetical decision‐making may differ from patients with a confirmed diagnosis. Last, our study did not stratify participants by the intensity of probable NeuP, an attribute that could contribute to the heterogeneity of treatment preferences.

In conclusion, the high burden of probable NeuP is significantly compounded by poor symptom recognition, leading to low treatment‐seeking rates and underdiagnosis. Given the potential for large‐scale, sustainable screening using this community‐based self‐assessment interface, future research should prioritize its diagnostic validation and implementation effectiveness. Extrapolating from our findings regarding the treatment preference of the general public, we suggest that an aversion to side effects predominantly dictates these preferences. Hence, effective patient‐provider communication remains the cornerstone of long‐term and sustainable care, particularly for chronic NeuP.

## Author Contributions

**Wee‐Kiat Tan:** data curation, formal analysis, investigation, methodology, software, visualization, writing – original draft. **Maznah Dahlui:** writing – review and edit. **Azhari Rosman:** investigation, writing – review and edit. **Hiang Leng Tan:** investigation, writing – review and edit. **Noraidah Guntalib:** investigation, writing – review and edit. **Siew Pheng Chan:** investigation, writing – review and edit. **Shalini Sree Dharan:** investigation, writing – review and edit. **Pei Chiek Teh:** investigation, writing – review and edit. **Daniel Yi Jie Wong:** investigation, writing – review and edit. **Kenny Tan:** investigation, writing – review and edit. **Solomon Tesfaye:** methodology, investigation, writing – review and edit. **Lee‐Ling Lim:** conceptualization, funding acquisition, methodology, supervision, writing – review and edit.

## Funding

This study was supported by the UMConsult Grant and Universiti Malaya (IF024‐2025). The funder has no role in study design, data collection, analysis, and manuscript writing.

## Conflicts of Interest

L.L.L. has received research grants via her institution from Abbott Diabetes Care, AstraZeneca, and Novartis and speaker honoraria from Abbott, AstraZeneca, Boehringer Ingelheim, Novo Nordisk, Roche Diabetes Care, and Zuellig Pharma. S.P.C. has received research grants from Zuellig Pharma, Novo Nordisk via her professional society (Malaysian Endocrine and Metabolic Society) and speaker honoraria from AstraZeneca, Abbott, Merck Serono, Novo Nordisk, Servier, and Zuellig Pharma. S.T. reports honoraria for educational meetings from Worwag Pharma, Novo Nordisk, Viatris, Merck, Nevro, P&G Health, Viatris, Medtronic, and Grunenthal; consulting fees from Worwag Pharma, Grunenthal, Merz, unrestricted research grants from Viatris, P&G Health, and Withings.

## Supporting information


**Data S1:** jns70154‐sup‐0001‐supinfo.docx.
**Figure S1:** Location of the nine (9) automated self‐assessment interfaces for community screening across Peninsular Malaysia.
**Figure S2:** An example of the automated self‐assessment interface used for community screening at a tertiary hospital.
**Table S1:** Comparison of demographic distribution between the community‐screening cohort in the present study and the general population in the Malaysian National Census data.
**Table S2:** Attributes of NeuP treatment and their levels in the DCE cohort.
**Table S3:** An example of a DCE choice task given to participants.
**Table S4:** The frequency of probable NeuP symptoms by gender, age, comorbidity status, and geographical regions in the community screening cohort in Malaysia.
**Table S5:** Ability to recognize probable NeuP symptoms in the community screening cohort.
**Table S6:** The tradeoffs between attributes using high risk of common side effects (10%) as the reference in the DCE cohort.
**Table S7:** Willingness‐to‐pay (WTP) for all levels of treatment attributes in the DCE cohort.
**Table S8:** Deterministic Heterogeneity: Interaction of attributes with the highest level of education, condition of pain, age, household income level, and education level in the DCE cohort.

## Data Availability

Data cannot be shared publicly due to local regulations imposed by the Medical Research Ethics Committee (MREC), Universiti Malaya Medical Centre (UMMC), as well as by the Government of Malaysia, effective from 29 April 2025 (https://www.pdp.gov.my/ppdpv1/wp‐content/uploads/2025/05/BUKU‐GARIS‐PANDUANPEMINDAHAN‐DATA‐PERIBADI‐RENTASSEMPADAN‐CBPDT.pdf). Researchers who are interested and meet the criteria for research access to our anonymized data may apply directly to Prof. Dr. Lee‐Ling Lim (limleeling@um.edu.my) or MREC UMMC (ummc-mrec@ummc.edu.my).
