# Supplementary material for: Burden and Treatment Preference of Probable Neuropathic Pain in 13 592 people: A Cross‐Sectional and Discrete Choice Experiment in the General Population in Malaysia
Source: J Peripher Nerv Syst. 2026 Aug 4;31(3):e70154. doi: 10.1111/jns.70154 (PMC13437251; doi:10.1111/jns.70154)
Supplement: Supplementary file 1 [file JNS-31-0-s001.docx]

**Supplementary Material**

*Table of Contents*

[Supplementary Information S1. Statistical modelling on the preference for anti-neuropathic agent.	2](#_Toc236570303)

[Supplementary Fig S1. Location of the nine (9) automated self-assessment interfaces for community screening across Peninsular Malaysia	3](#_Toc236570304)

[Supplementary Fig S2. An example of the automated self-assessment interface used for community screening at a tertiary hospital	4](#_Toc236570305)

[Supplementary Table S1. Comparison of demographic distribution between the community-screening cohort in the present study and the general population in the Malaysian National Census data.	5](#_Toc236570306)

[Supplementary Table S2. Attributes of NeuP treatment and their levels in the DCE cohort	6](#_Toc236570307)

[Supplementary Table S3. An example of a DCE choice task given to participants	7](#_Toc236570308)

[Supplementary Table S4. The frequency of probable NeuP symptoms by gender, age, comorbidity status, and geographical regions in the community screening cohort in Malaysia	8](#_Toc236570309)

[Supplementary Table S5. Ability to recognize probable NeuP symptoms in the community screening cohort	11](#_Toc236570310)

[Supplementary Table S6. The tradeoffs between attributes using high risk of common side effects (10%) as the reference in the DCE cohort	12](#_Toc236570311)

[Supplementary Table S7. Willingness-to-pay (WTP) for all levels of treatment attributes in the DCE cohort	13](#_Toc236570312)

[Supplementary Table S8. Deterministic Heterogeneity: Interaction of attributes with the highest level of education, condition of pain, age, household income level, and education level in the DCE cohort.	14](#_Toc236570313)

# **Supplementary Information S1**. Statistical modelling on the preference for anti-neuropathic agent.

Our analysis was grounded in random utility theory, which assumes that people select the option that provides the highest perceived utility. It is operationalized mathematically as below:

$$U_{nj}= V_{nj}+ \varepsilon_{nj}= \sum_{k} \beta_{k} \chi_{jk}+ \varepsilon_{nj}$$

Where $U_{nj}$ is the utility derived from participants (n) for choosing the choice (j), which comprises the deterministic component $(V_{nj})$ and the stochastic component ${(\varepsilon}_{nj})$. $\beta_{k}$ is the parameter to be estimated, $\chi_{jk}$ is the k^th^ attribute level of choice j. In the present DCE analysis, we defined the utility function as follows, where ASC is the alternative specific constant of the treatment option:

$$V_{nj}+\varepsilon_{nj}= {ASC}_{treatment}+\beta_{1}*{Pain Reduction}_{20-30\%}+ \beta_{2}*{Pain Reduction}_{30-50\%}+\beta_{3}*{Pain Reduction}_{>50\%}+ \beta_{4}*{Effect Time}_{2-3wk}+\beta_{5}*{Effect Time}_{>3wk}+\beta_{6}{*Proven Effectiveness}_{medium}+{\beta_{7}*Proven Effectiveness}_{high}+\beta_{8}*{Side Effect}_{medium}+\beta_{9}*{Side Effect}_{high}+\beta_{10}*Cost$$

The random estimation followed a type 1 extreme value error distribution on $\varepsilon_{nj}$. We used a multinomial logit (MNL) model to estimate a basic model. Subsequently, we used a mixed logit (MXL) model to explore heterogeneity of treatment preferences, accounting for both observed (deterministic) and unobserved (random) parameters. We initially specified a main-effects model with randomly distributed parameters, followed by models incorporating deterministic interactions between attribute levels and participants’ characteristics to further explore observed heterogeneity. All attributes were modelled as categorical variables, except for cost, which was specified as a continuous variable. We simulated random parameters using 300 draws generated through Modified Latin Hypercube Sampling to improve the stability of estimates.

We examined heterogeneity in preferences across all attribute levels. Given the known sensitivity of cost in DCE studies, we assessed the cost attribute using the linear, quadratic, and exponential models to identify the most behaviourally plausible specification. Last, we modelled the cost parameter using a negatively constrained log-normal distribution. Given a full covariance MXL was specified, the variance and choice trend induced by attribute non-attendance has been accommodated by the model through internal correlation of utility coefficients.

***Rationale for model choice:***

Although the MNL model is an important foundational model, it assumes no correlation between alternatives (restricted by Independence of Irrelevant Alternatives), which limits its interpretability in modelling human behavior. Latent class analysis (LCA) is useful to reveal hidden subgroups; however, it depends heavily on the assumption of local independence, which may limit its capability in managing attribute non-attendance or scale heterogeneity. In contrast, a mixed logit model (MXL) allows preference and its heterogeneity to vary across individuals continuously without predetermined discrete classifications. Hence, we adopted a mixed logit model in our final analysis to capture the spectrum of heterogeneity.

# **Supplementary Fig S1**. Location of the nine (9) automated self-assessment interfaces for community screening across Peninsular Malaysia

Footnote: The nine (9) automated self-assessment interfaces were distributed in Penang, Perak, and Kuala Lumpur. These were the tertiary hospitals that had very high traffic; hence, the locations were ideal for large-scale probable NeuP screening.

# **Supplementary Fig S2**. An example of the automated self-assessment interface used for community screening at a tertiary hospital

Footnote: The device occupies a small space, visually attractive to patients or family members who are waiting to see doctors. The screening project was a patient-centric initiative in collaboration with Viatris Malaysia. The interface calculates the ID-Pain score with pre-programmed algorithm according to ID-Pain questionnaire. The operating hour of the device is pre-preprogrammed (a timed on/off schedule), thus the operation is entirely automatic without the need of manpower.

# **Supplementary Table S1**. Comparison of demographic distribution between the community-screening cohort in the present study and the general population in the Malaysian National Census data.

| **Characteristics** | | **Malaysian National Census Data (general population)** | | **Community-screening cohort in the present study** | | **Difference** |
| --- | --- | --- | --- | --- | --- | --- |
| **Age groups** |  | **N** | **%** | **N** | **%** | **%** |
|  | 18-30 | 8,769,450 | 27 | 3,135 | 23 | 4 |
|  | 31-40 | 11,276,748 | 35 | 2,785 | 21 | 14 |
|  | 41-50 | 5,228,804 | 16 | 2,746 | 20 | 4 |
|  | 51-60 | 3,634,110 | 11 | 2,243 | 17 | 6 |
|  | 61-70 | 2,246,718 | 7 | 2,673 | 19 | 8 |
|  | 71-80 | 1,013,957 | 3 |  |  |  |
|  | >80 | 320,227 | 1 |  |  |  |
| **Gender** | Male | 17,957,700 | 52 | 6,493 | 48 | 4 |
|  | Female | 16,273,900 | 48 | 7,099 | 52 | 4 |
| **People living with diabetes** | | 5,340,145 | 15 | 2,331 | 17 | 2 |

Footnotes: To evaluate the generalizability of the community-screening cohort relative to the general population in Malaysia, we compared the distribution of participants' age, gender, and diabetes status, which are risk factors for NeuP, against the national census data. Apart from the under-representation of participants aged 31-40, other characteristics of the community-screening cohort demonstrated distributions similar to those of the general population of Malaysia (<10% difference), suggesting appropriate representation.

# **Supplementary Table S2**. Attributes of NeuP treatment and their levels in the DCE cohort

| **Attributes** | **Level** |
| --- | --- |
| **Perceived Pain Reduction** | <20% |
|  | 20-30% |
|  | 30-50% |
|  | >50% |
| **Time to Effect** | 1-2 weeks |
|  | 2-3 weeks |
|  | >3 weeks |
| **Proven Effectiveness** | Limited Scientific Evidence |
|  | Some Scientific Evidence |
|  | Strong Scientific Evidence |
| **Risk of Common Side Effects** | Low (0.001%) |
|  | Moderate (0.01%) |
|  | High (10%) |
| **Cost per tablet** | MYR 1 |
|  | MYR 2 |
|  | MYR 3 |
|  | MYR 4 |

Footnote: Five main attributes were identified through a consolidation of attributes from a systemic review and expert opinion. Within each attribute, several levels were identified. Subsequently, these attributes and their levels were used to build the survey questions. MYR: Ringgit Malaysia

# **Supplementary Table S3**. An example of a DCE choice task given to participants

Footnote: Each choice task provided 2 options (Treatment A and Treatment B) to participants. A total of 12 choice tasks with different scenarios were given to participants. DCE, discrete choice experiment.

# **Supplementary Table S4**. The frequency of probable NeuP symptoms by gender, age, comorbidity status, and geographical regions in the community screening cohort in Malaysia

| **Characteristics** | | **Overall community screening cohort** | | | | **ID Pain score ≥3 (Likely to have NeuP)** | | | | **ID-Pain score <3 (Unlikely to have NeuP)** | | | | |
| --- | --- | --- | --- | --- | --- | --- | --- | --- | --- | --- | --- | --- | --- | --- |
|  |  | N | Symptoms | Frequency | % | N | Symptoms | Frequency | % | N | Symptoms | Frequency | % |  |
| Gender | Men | 6,493 | Numbness | 2,722 | 41.92 | 1,189 | Pins and needles | 1,119 | 94.11 | 5,304 | Numbness | 1,650 | 31.11 |  |
|  |  |  | Pins and needles | 2,268 | 34.93 |  | Numbness | 1,072 | 90.16 |  | Pins and needle | 1,149 | 21.66 |  |
|  |  |  | Electric-shock | 1,946 | 29.97 |  | Electric-shock | 1,023 | 86.04 |  | Electric-shock | 923 | 17.40 |  |
|  |  |  | Hot/Burning | 1,518 | 23.38 |  | Hot/Burning | 934 | 78.55 |  | Hot/Burning | 584 | 11.01 |  |
|  |  |  | Made worse by touching clothing or bed sheets | 1,049 | 16.16 |  | Made worse by touching clothing or bed sheets | 788 | 66.27 |  | Made worse by touching clothing or bed sheets | 261 | 4.92 |  |
|  | Women | 7,099 | Numbness | 3,275 | 46.13 | 1,304 | Pins and needle | 1,204 | 92.33 | 5,795 | Numbness | 2,077 | 35.84 |  |
|  |  |  | Pins and needles | 2,703 | 38.08 |  | Numbness | 1,198 | 91.87 |  | Pins and needles | 1,499 | 25.87 |  |
|  |  |  | Electric-shock | 2,176 | 30.65 |  | Electric-shock | 1,097 | 84.13 |  | Electric-shock | 1,079 | 18.62 |  |
|  |  |  | Hot/Burning | 1,654 | 23.30 |  | Hot/Burning | 965 | 74.00 |  | Hot/Burning | 689 | 11.89 |  |
|  |  |  | Made worse by touching clothing or bed sheets | 1,024 | 14.42 |  | Made worse by touching clothing or bed sheets | 750 | 57.52 |  | Made worse by touching clothing or bed sheets | 274 | 4.73 |  |
| Age Group | 18-30 | 3,135 | Numbness | 1,224 | 39.04 | 624 | Pins and needle | 577 | 92.47 | 2,511 | Numbness | 654 | 26.05 |  |
|  |  |  | Pins and needles | 1,057 | 33.72 |  | Numbness | 570 | 91.35 |  | Pins and needles | 480 | 19.12 |  |
|  |  |  | Electric-shock | 1,011 | 32.25 |  | Electric-shock | 534 | 85.58 |  | Electric-shock | 477 | 19.00 |  |
|  |  |  | Hot/Burning | 730 | 23.29 |  | Made worse by touching clothing or bed sheets | 476 | 76.28 |  | Hot/Burning | 260 | 10.35 |  |
|  |  |  | Made worse by touching clothing or bed sheets | 612 | 19.52 |  | Hot/Burning | 470 | 75.32 |  | Made worse by touching clothing or bed sheets | 136 | 5.42 |  |
|  | 31-40 | 2,795 | Numbness | 1,101 | 39.39 | 404 | Pins and needles | 369 | 91.34 | 2,391 | Numbness | 735 | 30.74 |  |
|  |  |  | Pins and needles | 867 | 31.02 |  | Numbness | 366 | 90.59 |  | Pins and needles | 498 | 20.83 |  |
|  |  |  | Electric-shock | 776 | 27.76 |  | Electric-shock | 342 | 84.65 |  | Electric-shock | 434 | 18.15 |  |
|  |  |  | Hot/Burning | 488 | 17.46 |  | Hot/Burning | 291 | 72.03 |  | Hot/Burning | 197 | 8.24 |  |
|  |  |  | Made worse by touching clothing or bed sheets | 352 | 12.59 |  | Made worse by touching clothing or bed sheets | 247 | 61.14 |  | Made worse by touching clothing or bed sheets | 105 | 4.39 |  |
|  | 41-50 | 2,746 | Numbness | 1,201 | 43.74 | 398 | Pins and needles | 363 | 91.21 | 2,348 | Numbness | 846 | 36.03 |  |
|  |  |  | Pins and needles | 952 | 34.67 |  | Numbness | 355 | 89.20 |  | Pins and needle | 589 | 25.09 |  |
|  |  |  | Electric-shock | 744 | 27.09 |  | Electric-shock | 338 | 84.92 |  | Electric-shock | 406 | 17.29 |  |
|  |  |  | Hot/Burning | 560 | 20.39 |  | Hot/Burning | 296 | 74.37 |  | Hot/Burning | 264 | 11.24 |  |
|  |  |  | Made worse by touching clothing or bed sheets | 320 | 11.65 |  | Made worse by touching clothing or bed sheets | 198 | 49.75 |  | Made worse by touching clothing or bed sheets | 122 | 5.20 |  |
|  | 51-60 | 2,243 | Numbness | 1,090 | 48.60 | 379 | Pins and needle | 356 | 93.93 | 1,864 | Numbness | 746 | 40.02 |  |
|  |  |  | Pins and needle | 887 | 39.55 |  | Numbness | 344 | 90.77 |  | Pins and needles | 531 | 28.49 |  |
|  |  |  | Electric-shock | 659 | 29.38 |  | Electric-shock | 306 | 80.74 |  | Electric-shock | 353 | 18.94 |  |
|  |  |  | Hot/Burning | 508 | 22.65 |  | Hot/Burning | 271 | 71.50 |  | Hot/Burning | 237 | 12.71 |  |
|  |  |  | Made worse by touching clothing or bed sheets | 265 | 11.81 |  | Made worse by touching clothing or bed sheets | 182 | 48.02 |  | Made worse by touching clothing or bed sheets | 83 | 4.45 |  |
|  | >60 | 2,673 | Numbness | 1,381 | 51.66 | 688 | Pins and needles | 658 | 95.64 | 1,985 | Numbness | 746 | 37.58 |  |
|  |  |  | Pins and needles | 1,208 | 45.19 |  | Numbness | 635 | 92.30 |  | Pins and needles | 550 | 27.71 |  |
|  |  |  | Electric-shock | 932 | 34.87 |  | Electric-shock | 600 | 87.21 |  | Electric-shock | 332 | 16.73 |  |
|  |  |  | Hot/Burning | 886 | 33.15 |  | Hot/Burning | 571 | 82.99 |  | Hot/Burning | 315 | 15.87 |  |
|  |  |  | Made worse by touching clothing or bed sheets | 524 | 19.60 |  | Made worse by touching clothing or bed sheets | 435 | 63.23 |  | Made worse by touching clothing or bed sheets | 89 | 4.48 |  |
| Comorbidity | Diabetes | 2,331 | Numbness | 1,496 | 64.18 | 935 | Pins and needles | 898 | 96.04 | 1,396 | Numbness | 618 | 44.27 |  |
|  |  |  | Pins and needles | 1,397 | 59.93 |  | Numbness | 878 | 93.90 |  | Pins and needles | 499 | 35.74 |  |
|  |  |  | Electric-shock | 1,072 | 45.99 |  | Electric-shock | 807 | 86.31 |  | Electric-shock | 265 | 18.98 |  |
|  |  |  | Hot/Burning | 1,020 | 43.76 |  | Hot/Burning | 787 | 84.17 |  | Hot/Burning | 233 | 16.69 |  |
|  |  |  | Made worse by touching clothing or bed sheets | 740 | 31.75 |  | Made worse by touching clothing or bed sheets | 653 | 69.84 |  | Made worse by touching clothing or bed sheets | 87 | 6.23 |  |
| Geographic Region | Northern Region | 9,056 | Numbness | 3,902 | 43.09 | 1,709 | Pins and needles | 1,599 | 93.56 | 7,347 | Numbness | 2,353 | 32.03 |  |
|  |  |  | Pins and needles | 3,270 | 36.11 |  | Numbness | 1,549 | 90.64 |  | Pins and needles | 1,671 | 22.74 |  |
|  |  |  | Electric-shock | 2,784 | 30.74 |  | Electric-shock | 1,439 | 84.20 |  | Electric-shock | 1,354 | 18.31 |  |
|  |  |  | Hot/Burning | 2,195 | 24.24 |  | Hot/Burning | 1,359 | 79.52 |  | Hot/Burning | 836 | 11.38 |  |
|  |  |  | Made worse by touching clothing or bed sheets | 1,562 | 17.25 |  | Made worse by touching clothing or bed sheets | 1,163 | 68.05 |  | Made worse by touching clothing or bed sheets | 399 | 5.43 |  |
|  | Central Region | 4,441 | Numbness | 2,060 | 46.39 | 768 | Pins and needles | 711 | 92.58 | 3,673 | Numbness | 1,355 | 36.89 |  |
|  |  |  | Pins and needles | 1,666 | 37.51 |  | Numbness | 705 | 91.80 |  | Pins and needles | 955 | 26.00 |  |
|  |  |  | Electric-shock | 1,313 | 29.57 |  | Electric-shock | 666 | 86.72 |  | Electric-shock | 647 | 17.62 |  |
|  |  |  | Hot/Burning | 961 | 21.64 |  | Hot/Burning | 532 | 69.27 |  | Hot/Burning | 429 | 11.68 |  |
|  |  |  | Made worse by touching clothing or bed sheets | 500 | 11.26 |  | Made worse by touching clothing or bed sheets | 366 | 47.66 |  | Made worse by touching clothing or bed sheets | 134 | 3.65 |  |

Footnote: The subgroups of age group, gender, comorbidity, and geographic regions were compared between overall community screening cohort, participants who likely had pain (ID Pain score ≥3), and participants who unlikely to have pain (ID Pain score <3). Participants with probable pain consistently complained of pins and needles, numbness, and electric-shock across all subgroups. Whereas participants without probable pain complained mostly numbness, followed by pins and needles and electric-shock. NeuP, neuropathic pain.

# **Supplementary Table S5**. Ability to recognize probable NeuP symptoms in the community screening cohort

| **Ability to recognize probable NeuP symptoms** | **N** | **%** |
| --- | --- | --- |
| Very good | 0.0 | 0.0 |
| Good | 715.0 | 17.8 |
| Moderate | 2201.0 | 55.0 |
| Poor | 1083.0 | 27.2 |

Footnote: The ability to recognize probable NeuP symptoms was defined by the composite score for correctly differentiating neuropathic symptoms from nociceptive symptoms. Most participants had poor to moderate ability to recognize probable NeuP symptoms. None of the participants had a very good ability to recognize probable NeuP symptoms.

# **Supplementary Table S6**. The tradeoffs between attributes using high risk of common side effects (10%) as the reference in the DCE cohort

| **Reference** | **Attributes** | **Tradeoff unit** |
| --- | --- | --- |
| High risk of side effects (10%) | Strong scientific evidence | 1.40 |
|  | >50% perceived pain reduction | 1.58 |
|  | Moderate risk of side effects | 3.22 |
|  | Some scientific evidence | 4.00 |
|  | 30-50% perceived pain reduction | 5.26 |
|  | >3 weeks time to effect | 5.88 |
|  | 2-3 weeks time to effect | 9.09 |

Footnotes: The tradeoff unit represents the required increase in a specific attribute for participants to accept a high (10%) risk of side effects. For example, to tolerate this risk, participants required a 1.58-unit increase in pain reduction efficacy (relative to a baseline of 50% perceived pain reduction). DCE, discrete choice experiment.

# **Supplementary Table S7**. Willingness-to-pay (WTP) for all levels of treatment attributes in the DCE cohort

| **Attributes** | **Levels** | **Mean WTP (MYR)** | **95% CI** |  |
| --- | --- | --- | --- | --- |
| Perceived Pain Reduction | <20% | Reference | - |  |
|  | 20-30% | 0.870 | -0.161, 1.901 |  |
|  | 30-50% | 1.741 | 0.253, 3.228 |  |
|  | >50% | 9.350 | 3.611, 15.088 |  |
| Time to Effect | 1-2 weeks | Reference | - |  |
|  | 2-3 weeks | -0.534 | -1.245, 0.177 |  |
|  | >3 weeks | 0.715 | -0.170, 1.600 |  |
| Level of Scientific Evidence | Limited scientific evidence | Reference | - |  |
|  | Some scientific evidence | 2.400 | 0.535, 4.270 |  |
|  | Strong scientific evidence | 11.630 | 4.731, 18.534 |  |
| Risk of Common Side Effects | Low (0.001%) | Reference | - |  |
|  | Moderate (0.01%) | -6.023 | -9.517, -2.528 |  |
|  | High (10%) | -18.662 | -29.943, -7.380 |  |

Footnotes: A positive value indicates the WTP for an improvement in an attribute relative to the reference level, whereas a negative value represents the WTP to avoid an unfavorable attribute. For example, participants were willing to pay MYR 11.630 for strong scientific evidence and MYR 18.66 to avoid a high risk of common side effects. From a health equity perspective, it is important to note that WTP measures the perceived monetary value of an attribute exchange, which does not necessarily reflect the participants' actual ability to pay (affordability). CI, confidence interval.

# **Supplementary Table S8**. Deterministic Heterogeneity: Interaction of attributes with the highest level of education, condition of pain, age, household income level, and education level in the DCE cohort.

| **Attributes** | | | **Levels** | | | **Level of education** | | | | | **Condition of probable NeuP** | | | |
| --- | --- | --- | --- | --- | --- | --- | --- | --- | --- | --- | --- | --- | --- | --- |
|  |  |  |  |  |  | **High education** | | | **Low education** | | **Probable NeuP** | | **Without probable NeuP** | |
|  |  |  |  |  |  | **Coefficient** | | **95% CI** | **Coefficient** | **95% CI** | **Coefficient** | **95% CI** | **Coefficient** | **95% CI** |
| Cost | | | Cost/tablet | | | -0.504 | | -6.219, -3.861 | -5.980 | -7.273, -4.686 | -5.604 | -6.852, -4.357 | -5.715 | -7.221, -4.209 |
| Perceived Pain Reduction | | | >50% | | | 1.120 | | 1.054, 1.187 | 0.997 | 0.887, 1.106 | 1.120 | 1.054, 1.187 | 1.011 | 0.919, 1.103 |
| Time to Effect | | | >3 weeks | | | -0.290 | | -0.332, -0.249 | -0.290 | -0.332, -0.249 | -0.290 | -0.332, -0.249 | -0.290 | -0.332, -0.249 |
| Level of Scientific Evidence | | | Strong scientific evidence | | | 1.289 | | 1.223, 1.354 | 1.000 | 0.895, 1.106 | 1.238 | 1.170, 1.306 | 1.191 | 1.100, 1.282 |
| Risk of Common Side Effects | | | High (10%) | | | -1.799 | | -1.890, -1.708 | -1.586 | -1.743, -1.429 | -1.811 | -1.908, -1.714 | -1.631 | -1.759, -1.503 |
|  |  | | | **Age (years)** | | | | | | | | | | |
|  | | |  | | | **Age (<30)** | | | **Age (31-50)** | | **Age (51-70)** | | **Age (>70)** | |
|  | | |  | | | **Coefficient** | | **95% CI** | **Coefficient** | **95% CI** | **Coefficient** | **95% CI** | **Coefficient** | **95% CI** |
| Cost | | | Cost/tablet | | | -5.763 | | -7.007, -4.519 | -5.756 | -7.302, -4.210 | -5.854 | -7.110, -4.599 | -5.727 | -6.989, -4.465 |
| Perceived Pain Reduction | | | >50% | | | 1.011 | | 0.919, 1.103 | 1.011 | 0.919, 1.103 | 1.265 | 1.149, 1.380 | 1.549 | 1.153, 1.946 |
| Time to Effect | | | >3 weeks | | | -0.290 | | -0.332, -0.249 | -0.290 | -0.332, -0.249 | -0.290 | -0.332, -0.249 | -0.290 | -0.332, -0.249 |
| Level of Scientific Evidence | | | Strong scientific evidence | | | 1.289 | | 1.192, 1.385 | 1.227 | 1.152, 1.302 | 1.130 | 1.023, 1.236 | 1.204 | 0.860, 1.545 |
| Risk of Common Side Effects | | | High (10%) | | | -1.706 | | -1.843, -1.570 | -1.706 | -1.843, -1.570 | -1.896 | -2.051, -1.741 | -2.511 | -3.065, -1.957 |
|  | |  | | |  | | **Household income level** | | | | | |  |  |
|  | | |  | | | **Low income (<MYR 5,000)** | | | **Middle income (MYR 5,000 – 10,000)** | | **High income (>MYR 10,000)** | |  |  |
|  | | |  | | | **Coefficient** | | **95% CI** | **Coefficient** | **95% CI** | **Coefficient** | **95% CI** |  |  |
| Cost | | | Cost/tablet | | | -5.658 | | -6.911, -4.406 | -5.742 | -6.998, -4.487 | -5.729 | -7.008, -4.451 |  |  |
| Perceived Pain Reduction | | | >50% | | | 0.943 | | 0.858, 1.028 | 1.011 | 0.919, 1.103 | 1.282 | 1.173, 1.391 |  |  |
| Time to Effect | | | >3 weeks | | | -0.290 | | -0.332, -0.249 | -0.290 | -0.332, -0.249 | -0.290 | -0.332, -0.249 |  |  |
| Level of Scientific Evidence | | | Strong scientific evidence | | | 1.110 | | 1.028, 1.192 | 1.326 | 1.238, 1.414 | 1.256 | 1.157, 1.355 |  |  |
| Risk of Common Side Effects | | | High (10%) | | | -1.549 | | -1.668, -1.430 | -1.821 | -1.941, -1.701 | -1.958 | -2.100, -1.816 |  |  |

Footnotes: The heterogeneity of interaction with participants characteristics was observed only at the highest level of respective attributes. Participants with higher education, household income and probable pain showed more aversion against high risk of side effects than their counterparts. However, higher educated participants showed lower cost sensitivity on the cost per tablet. Older participants (Age group 50 - 70 & >70 years old) had stronger preference on perceived pain reduction compared to the younger groups. DCE, discrete choice experiment. MYR, Malaysian Ringgit. CI, confidence interval.
